# Clothianidin and Thiacloprid Mixture Administration Induces Degenerative Damage in the Dentate Gyrus and Alteration in Short-Term Memory in Rats

**DOI:** 10.1155/2021/9983201

**Published:** 2021-11-23

**Authors:** Alejandra Mora-Gutiérrez, Jorge Guevara, Carmen Rubio, Minerva Calvillo-Velasco, Daniela Silva-Adaya, Socorro Retana-Márquez, Blanca Espinosa, Carmen Martínez-Valenzuela, Moisés Rubio-Osornio

**Affiliations:** ^1^Departamento de Neuroquímica, Instituto Nacional de Neurología y Neurocirugía, Ciudad de México 14269, Mexico; ^2^Departamento de Bioquímica, Facultad de Medicina, Universidad Nacional Autónoma de México, Ciudad de México 04510, Mexico; ^3^Departamento de Neurofisiología, Instituto Nacional de Neurología y Neurocirugía, Ciudad de México 14269, Mexico; ^4^Laboratorio Experimental de Enfermedades Neurodegenerativas, Instituto Nacional de Neurología y Neurocirugía, 14269 Ciudad de México, Mexico; ^5^Departamento de Biología de la Reproducción, Laboratorio R012, Universidad Autónoma Metropolitana, Unidad Iztapalapa, Ciudad de México 09340, Mexico; ^6^Departamento de Bioquímica, Instituto Nacional de Enfermedades Respiratorias, ICV, Ciudad de México 14080, Mexico; ^7^Unidad de Investigación en Ambiente y Salud, Universidad Autónoma de Occidente, Los Mochis, SIN, Mexico

## Abstract

Neonicotinoids are pesticides that act as agonists of nicotinic receptors for acetylcholine in insects' central nervous system (CNS). Chronic exposure to neonicotinoids in humans is related to autism, memory loss, and finger tremor. In this article, we evaluate the effect of subchronic oral administration of two neonicotinoids in the same mixture: clothianidin and thiacloprid. Decreasing doses of both pesticides were administered to rats starting from the lethal dose 50 (LD_50_) reported by the manufacturer. Our results indicate that the administration of three doses of decreasing amounts of LD_50_ (5/10, 4/10, and 3/10 LD_50_) resulted in 100% death in all cases. Ten administration times of 2/10 LD_50_ of the mixture caused only 20% of death cases after twenty-seven days, which was determined as a subchronic administration scheme. The animals administered 2/10 LD_50_ showed behavioral alterations after the first and second administration. Electrographic studies showed abnormal discharge patterns in the CNS. 72 h after the tenth dose, learning and memory tests were performed in the Morris water maze. Our results revealed significant decreases in permanence at the quadrant and the number of crosses (*P*=0.0447,  *P*=0.0193, respectively), which represent alterations in the short-term memory test, but there were no significant changes in a long-term memory test. Likewise, the brains of these animals showed tissue architecture loss, nucleosomal retraction, and a significant increase in the pycnosis of the granular neurons of the dentate gyrus analyzed at 72 h after the last dose (*P*=0.0125). Toxic effects and cognitive deterioration that have been found in communities living near contaminated areas are probably related to the agricultural use of neonicotinoids.

## 1. Introduction

Neonicotinoids are the last generation of insecticides used in agriculture for the protection of crops against harmful insects. These are derived from nicotine and are classified as N-nitroguanidines (clothianidin, imidacloprid, thiamethoxam, and dinotefuran) and N-cyano-aminides (thiacloprid and acetamiprid) [1]. Neonicotinoids act as agonists of the nicotinic receptors for acetylcholine (nAChRs) in insects and mammals, especially on the subtype *α*4*β*2. In Mexico, these kinds of insecticides are used to treat seeds, soil, or leaves of crops such as cotton, chili, tomato, potato, tobacco, corn, and apple, among others, including ornamental plants [2].

Mixtures of pesticides are currently used in agriculture to produce rapid destruction of pests or the economy of carrying out a single spraying operation [3]. Since the use of mixtures of pesticides is common and excessive, there is currently concern about their impact on human health, both by the workers who produce and employ the mixtures and by the contamination of food and water [4]. Recently, it has been found that insecticide mixtures are related to neurotoxic effects [5, 6].

Although there are reports of accidental acute poisoning caused by dermal contact, inhalation, or ingestion of imidacloprid, acetamiprid, and thiacloprid, in which signs such as muscle weakness, convulsions, drowsiness, dyspnea, mydriasis, abdominal cramps, and muscle spasms were observed, the harmful effects of neonicotinoids on human CNS are still to be clarified [6–8]. These signs are similar to those presented by experimental animals [9–11]. In humans, chronic exposure to neonicotinoid pesticides has been linked to neurological and developmental problems, such as tetralogy of Fallot, anencephaly, and autistic spectrum disorder, and symptoms such as memory loss and finger tremor [12].

Furthermore, usage of neonicotinoid pesticides alone or in mixtures is increasing and in 2014 represented about 25% of the global insecticide market [13]. It is necessary to investigate the potential toxic effect these pesticides can have on other types of organisms. The neurotoxic effect in the brain caused by neonicotinoids and their repercussions on mammal memory and learning, due to prolonged exposure, is not yet clear, and the available information obtained is from studies of individual neonicotinoids [14–16].

In this regard, the goal of this study is to evaluate the effect of subchronic administration of a mixture of two neonicotinoid pesticides (clothianidin and thiacloprid) on the processes of learning and memory, as well as its effect on the hippocampal tissue of rats.

## 2. Materials and Methods

### 2.1. Animals

The adult male Wistar rats (300–320 g) used throughout the study were provided by the animal research facilities at the Faculty of Medicine at the National Autonomous University of Mexico. All experimental procedures were carried out following the NIH Guidelines for the Care and Use of Laboratory Animals. The animals were housed in transparent acrylic boxes with a temperature of 20°–22°C, 15 air changes per hour, and a photoperiod of 12 h light/12 h dark. The rats were fed with a rodent diet and purified water ad libitum. Neonicotinoid pesticides manufactured by Bayer CropScience Mexico Laboratories were used in the present study from two commercial brands: Poncho 600 (clothianidin, 600 g/L) and Calypso 480 (thiacloprid, 480 g/L). The lethal doses 50 (LD_50_) reported by the manufacturer in rats are >2000 mg/kg and >300–<500 mg/kg for clothianidin and thiacloprid, respectively (Safety Data Sheets from Bayer CropScience Laboratories, 2005).

### 2.2. Neonicotinoids Administration Scheme

In order to corroborate the LD_50_ of clothianidin and thiacloprid effect reported by the manufacturer, two groups of rats (*n* = 10) were initially orally administered commercial brands of LD_50_ (2000 mg/kg and 400 mg/kg, respectively). Likewise, to determine the minimum dose to be used in a subchronic administration scheme, four groups of rats (*n* = 10) were administered decreasing doses of the clothianidin and thiacloprid mixture, starting from 50% LD_50_: first group, 5/10 LD_50_, 1000 mg/kg of clothianidin plus 200 mg/kg of thiacloprid; second group, 4/10 LD_50_, 800 mg/kg plus 160 mg/kg; third group, 3/10 LD_50_, 600 mg/kg plus 120 mg/kg; and fourth group, 2/10 LD_50_, 400 mg/kg plus 80 mg/kg. The fifth group of animals (*n* = 10) was administered physiological saline solution (SSF) and was considered as a control group. The different doses of pesticides were diluted 1 : 5, for each 100 mL of pesticide, to which 400 mL of SSF were added. Once diluted, they were kept separately in sterile containers and mixed at the moment of administration, which was carried out orally employing a forced feeding cannula. The animals were administered one hour after the start of their light cycle and were kept under observation for 4 hours after administration. During this time, animal signs in response to each dose were recorded, and mortality was registered. The animals with a mixture of 2/10 LD_50_, equal to 400 mg/kg of clothianidin plus 80 mg/kg of thiacloprid, were subjected to a subchronic administration scheme, every 72 h for 27 days.

### 2.3. Electrophysiological Records

Throughout this paper, the objective is to demonstrate the electrophysiological activities produced at the CNS level due to the neonicotinoid mixture administration of 400 mg/kg of clothianidin plus 80 mg/kg of thiacloprid. The rats (*n* = 3) were anesthetized with intramuscular Zoletil® 50 (30 mg/kg) and placed on a stereotaxic apparatus to connect electrodes for the recording of electroencephalographic (EEG) activity in the left sensory-motor cortex. Each stainless-steel wire electrode was joined to a screw collocated on each rat skull. Additionally, another electrode was used as an independent source of reference. Electrodes were arranged and secured to the skull with dental acrylic, and skin cuts were sutured while exposing the electrodes. Seven days after postoperative recovery and 1 hour after neonicotinoid mixture administration, the rats were placed in a chamber while their electrodes were connected to a digital EB neuropolygraph via flexible cables. The electrographic activity was recorded for 1 hour for each rat. Data was stored and digitized on a PC with software provided by Stellate Systems.

### 2.4. Morris Water Maze Spatial-Reference Task

The Morris water maze (MWM) was run as previously described [17]. The apparatus consists of a platform of transparent acrylic (19 cm in diameter and 22 cm in height) set inside a circular water pool (170 cm in diameter and 50 cm in height), which was filled with water (21 ± 2°C) to a height of 30 cm. The platform was placed in a constant position, equidistant from the center and the edge of the pool wall and submerged 2 cm below the water surface, within one of the four (northeast) imaginary quadrants (northeast, northwest, southeast, and southwest) into which the pool was divided. Three figures were placed on the white walls around the tub, which functioned as visual-spatial clues. The fourth position was identified as the entrance door to the pool, which was between the southwest and southeast quadrants. For the present analysis, two groups of animals were used (*n* = 10); the first was subjected to the subchronic, neonicotinoid mixture administration scheme at a dose of 400 mg/kg of clothianidin plus 80 mg/kg of thiacloprid. The second group was administered volumes of SSF equivalent to those used in the first group. Subchronic administration was performed, and the animals were weighed every 72 h to correct the dosage. The weight loss did not exceed 20% of their initial body weight. 72 h after the last administration, rats were subjected to 5 learning sessions, which consisted of 4 trials per day for 4 consecutive days (days 1–4) and a single trial on day 6, in which the time, or latency, of arrival to the platform was measured. On day one and only in the first trial, each rat was placed in one of the four possible starting positions. If it did not find the platform in the first 60 seconds, it was guided to it and left there for 30 seconds to achieve spatial location. In the following tests, rats were released from the four different starting points, and if the platform was not found during the first 60 seconds, this time would be taken as the maximum score and the animal would be removed from the pool.

To evaluate the short-term memory (STM), on the 7th day after administration, a single trial was carried out, in which each animal was placed in 1 of the 4 possible starting positions in the absence of the platform. The number of crosses made to where the platform was located and the time spent in that quadrant were recorded. On the 13th day, the long-term memory (LTM) was evaluated. A single test was performed with the platform in place, in which each rat was released from one of the four possible starting positions; the arrival latency time was quantified [17].

### 2.5. Histology

19 days and 72 hours after the last test in the MWM, the animals were administered an overdose of intraperitoneal sodium pentobarbital and perfused with 200 mL of SSF, followed by 200 mL of paraformaldehyde at 4%. All brains were removed and postfixed with the same fixative solution until processing. All the brains were dehydrated in alcohol and xylol solutions at increasing concentrations and were then immersed in paraffin, and 5 *μ*m thick coronal sections were taken from each brain at the level of the dorsal hippocampus (Hp) (−3.8 to −4.3 mm posterior to bregma). Eight serial cuts were selected per rat, hydrated, and then stained for 1 hour with the cresyl violet dye. After 1 hour, they were dehydrated again and mounted. From each of the 8 serial sections, microphotographs of the CA1, CA2, CA3, and the dentate gyrus (DG) regions of the Hp were taken, with a 40X objective for cellular quantification of 250 linear *μ*m per region, both right and left hippocampus. For the counting of viable cells, only those with a well-defined nuclear membrane and prominent nucleoli were considered. To quantify the damaged cells, those that were observed as pyknotic cells were considered.

### 2.6. Statistical Analysis

The data obtained from MWM arrival latencies were analyzed using a one-way ANOVA variance analysis, followed by a Duncan post hoc test. Both percentages of permanence in the quadrant analysis and the number of crosses were analyzed with a one-way Student t-test, followed by a Mann–Whitney *U* test. The data obtained from the cell count were analyzed with the Student t-test. The differences were considered statistically significant for *P* < 0.05.

## 3. Results

### 3.1. Administration and Behavioral Changes of Neonicotinoids

To corroborate the LD_50_ administration effect, doses corresponding to the LD_50_ of clothianidin and thiacloprid reported by the manufacturer were administered individually. Our results showed 100% death (*n* = 10) in the animals administered 2000 mg/kg clothianidin (LD_50_). Likewise, we found 100% death (*n* = 10) in the animals administered an LD_50_ of 400 mg/kg of thiacloprid. Both results were obtained after the first dose. In the same way, in the group of animals (*n* = 10) administered the highest dose of 1000 mg/kg of clothianidin plus 200 mg/kg of thiacloprid (5/10 from LD_50_) used in this study, 100% death (*n* = 10) occurred after the first dose. Doses of 800 mg/kg clothianidin plus 160 mg/kg of thiacloprid (4/10 from LD_50_) (*n* = 10) and 600 mg/kg of clothianidin plus 120 mg/kg thiacloprid (3/10 from LD_50_) (*n* = 10) resulted in 100% mortality after three and five administration times, respectively (Table 1). Before death, the animals presented the following signs: decreased activity and ptosis, followed by slow gait and prostration, and previous myoclonus, as well as startles during prostration that raised some animals up to 10 centimeters from the box floor. In some instances, running moves and squeals were observed (Table 2). With time, the presence and absence of signs alternated, but after continuous episodes of anterior myoclonus and prostration, the animals developed dyspnea and seizures lasting a few seconds (approximately 10 seconds), in which they aggressively agitated in a generalized manner. Finally, death occurred 3 to 4 hours after administration in most registered cases; the remaining animals' deaths occurred during the night.

The animals of the group (*n* = 10) administered a mixture of 400 mg/kg of clothianidin plus 80 mg/kg of thiacloprid (2/10 from LD_50_) presented a set of signs very similar to those observed in the animals administered the highest doses. In these cases, there was a decrease in the consumption of food and water, decrease in motor activity, ptosis, slow gait, shrieks, previous myoclonus, prostration, and dyspnea, and in only 20% there were there seizures and death (Tables 1 and 2). The animals did not show more of these behavioral changes after the third administration. Consequently, we determined that a dose of 400 mg/kg of clothianidin plus 80 mg/kg of thiacloprid is a useful dose to establish the subchronic administration schedule.

### 3.2. Electrophysiological Records

**Figure 1:**
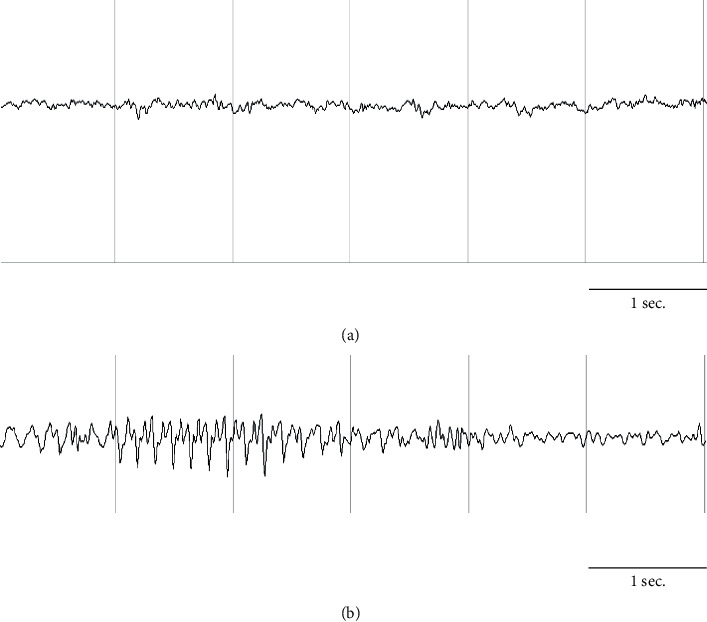
Representative electroencephalographic record of electrographic changes taken immediately after the last administration of the neonicotinoid mixture. (a) Record taken from control rats. (b) Rats administered a neonicotinoid mixture (LD_50_ 2/10) showing abnormal electrographic activity, characterized by spikes in amplitude and low frequency.

To determine behavioral alterations, we performed an electroencephalographic record. We recorded the sensorimotor cortex 1 hour after administration (*n* = 3) (a mixture of the low doses of two neonicotinoids). We found that the animals showed behavioral changes such as a fixed gaze, head oscillations, altered gait, forelimb clonus, and generalized seizures; these changes are associated with a record characterized by low-frequency rhythms such as theta and delta (Figure 1).

### 3.3. Effect of Administration of Neonicotinoid Mixture on Spatial Learning and Memory

**Figure 2:**
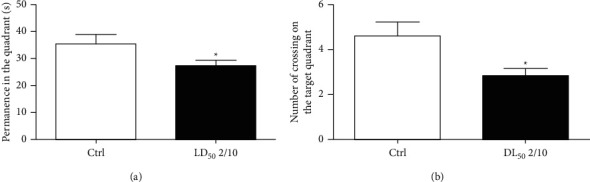
Effect of neonicotinoid mixture oral administration (LD_50_ 2/10) on the short-term memory of rats. (a) Animals' permanence time in the quadrant during the memory test. (b) Number of crossings on the target quadrant. Both parameters indicate impairment in the short-term memory test when compared with the control group. All values are given as mean ± SE (*n* = 10). ^*∗*^*P*=0.0447 (a) and ^*∗*^*P*=0.0193 (b).

72 hours after the last oral administration of neonicotinoid mixture, the animals were subjected to the MWM in 4 learning sessions. It consisted of 4 trials per day for 4 consecutive days (days 1–4) and a single trial on the 6th day, in which the latency of arrival to the platform was measured. Results of the learning tests in the MWM indicated that the latencies of arrival to the platform were 17.57 ± 5.34 s for rats administered a dose of 400 mg/kg of clothianidin plus 80 mg/kg of thiacloprid (*n* = 10), and they did not show statistically significant differences when compared with those of the control group (*n* = 10), with latency times of 15.38 ± 9.44 s (data not shown). The results of short-term memory analysis, evaluated 7 days after the last administration, showed significant differences in the time of permanence in the quadrant (*P* = 0.0447) (*n* = 10) compared with the control group rats (*n* = 10) (Figure 2(a)). Likewise, the number of crosses (4.6 ± 1.95 crosses/min) of the control group (Figure 2(b)) showed significant differences (*P*=0.0193) compared to that of the experimental group (2.8 ± 1.13 crosses/min). The long-term memory was evaluated on the 13th day. The average arrival latency was 8.66 ± 5.44 s for the control group and did not show statistical differences compared with that of the group administered neonicotinoid mixture (10.88 ± 5.50 s) (data not shown).

### 3.4. Histology

**Figure 3:**
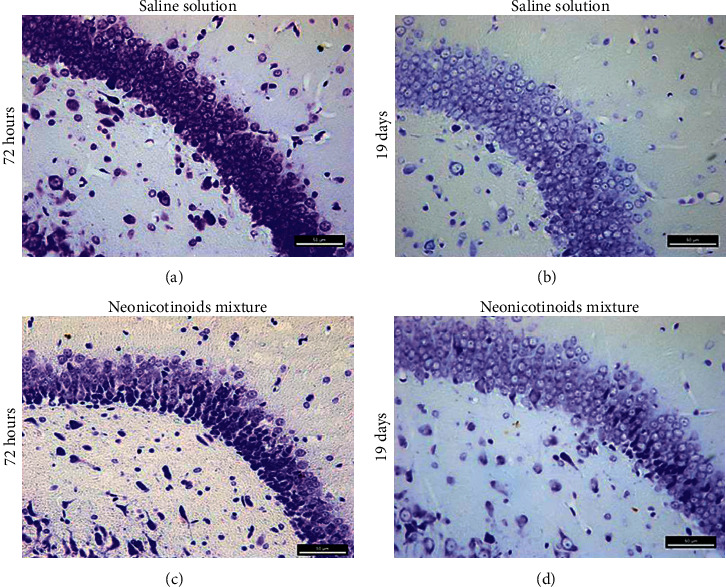
(a) Neuronal damage produced by the oral administration of the neonicotinoid mixture (LD50 2/10). (b) Effect of the oral administration of saline solution observed in a magnification of 40X, showing the granular cells of DG 72 h and 19 days after the proofs on MWM. (c) Evidence of tissue architecture loss, pyknotic cells, and nucleosomatic retraction on the granular cells of DG of the rat. (d) Evaluations carried out during the 19 days show minor damage caused by the neonicotinoid mixture administration.

**Figure 4:**
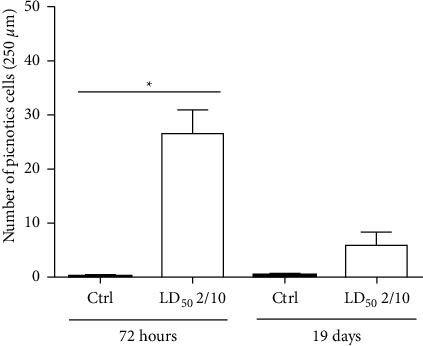
Cell count of pyknotic cells in the DG of rats administered the neonicotinoid mixture (LD_50_ 2/10), to which euthanasia was applied 72 h after the last dose. The pyknotic cells quantified were higher and statistically significant (*P*=0.0125) when compared to their respective control (*n* = 5). The DG cells from the rats killed 19 days after the last dose showed a less severe effect (*n* = 5).

The mixture administration of clothianidin and thiacloprid did not present any histological alteration in CA1, CA2, and CA3 hippocampal tissue regions (data not shown). 72 hours after the tenth administration of the neonicotinoid mixture (*n* = 5), the granular cells of the Hp at the level of the DG showed a significant increase (*P*=0.0125) of pycnosis cells. The DG cells from the rats sacrificed 19 days after the last pesticide administration, and later the MWM test showed a similar, but less histopathologically severe (*n* = 5), effect (Figures 3 and 4). Nucleosomatic retraction and light to moderate tissue architecture loss in the DG arm and the DG granular cells were also observed, with the granular cells of the ectal and endal arm of the DG being the most affected (Figure 3). All morphological changes observed were different compared to the control group (*n* = 5). The cell count did not show statistical differences for the CA1, CA2, and CA3 regions in any of the 4 different groups evaluated (Ctrl 72 h, neonicotinoid mixture 72 h, Ctrl 19 days, and neonicotinoid mixture 19 days).

## 4. Discussion

Neonicotinoid insecticides such as clothianidin and thiacloprid are first-generation, water-soluble compound pesticides [18, 19]. This chemical property limits these compounds to overpass the blood-brain barrier (BBB) [20]. In addition to the high selectivity of these pesticides on the nicotinic acetylcholine receptors (nAChRs) of insects, they have been given safety profiles favorable to humans [11]. However, in the present study, oral administration of the pesticide LD_50_ resulted in 100% death in the animals. Neonicotinoids have an agonist action and binding affinity for nicotinic cholinergic receptors, especially on the *α*4*β*2 and *α*7 subtype of vertebrates [21, 22]; death can be the result of cholinergic overstimulation. Neonicotinoids toxicity in this study was mainly observed by nervous signs; however, it has been reported that neonicotinoids such as clothianidin and thiacloprid in doses equivalent to 1/10 of the LD50 acutely administered can cause vascular disorders and consequently dystrophy and hemorrhage in parenchymal organs and hepatic necrosis, among other lesions, generating alterations, that could also contribute significantly to the death of animals [23]. This evidence cast doubt regarding the safety margins established for each pesticide and even its World Health Organization (WHO) toxicological classification. There is a possibility that the active constituents, clothianidin and thiacloprid, respectively, of the mixture of Poncho 600 and Calypso 480 exert a central effect on the nAChRs isoform *α*4*β*2 (the main targets of these pesticides in the cortex) [24, 25]. Other brain structures that regulate movement cannot be ruled out and are believed to be related to the motor signs observed, including myoclonus, tremor, ataxia, and seizures, with the oral administration of 5 mg/kg or intravenous administration of 1 mg/kg of thiacloprid radiolabeled with ^14^C-methylene after 1 hour and 5 minutes, respectively [9, 10, 26]. Some results indicated a significant increase in the pesticide signal within the CNS [27, 28]. Similarly, toxicokinetic studies with radiolabels demonstrate the presence of 0.338 *μ*g per gram of brain tissue analyzed, 1 hour after the oral administration of 5 mg/kg clothianidin in rats [29]. The intraperitoneal administration of 10 or 20 mg/kg of imidacloprid, acetamiprid, thiacloprid, and nitenpyram reaches maximum levels of 11 to 16 *μ*g of nitenpyram and thiacloprid, 6 *μ*g of imidacloprid, and 3 *μ*g of acetamiprid in the brain of rodents [28]. The above is evidence that neonicotinoids are capable of overcoming the BBB, which can lead to the animals' death, with a mixture of clothianidin plus thiacloprid administered orally, as demonstrated in the present study. Animal death with the highest doses of neonicotinoid administration is thought to be a consequence caused by a combination of both pesticides. In pilot experiments in our laboratory, when rats were administered the same dose individually, clothianidin (1000 mg/kg) caused the animals' death only after several doses (data not shown). In the case of thiacloprid (200 mg/kg), it was not possible to cause death in repeated doses (data not shown). However, it was possible when the animals were administered the mixture of clothianidin plus thiacloprid in a concentration of 800 mg/kg plus 160 mg/kg or 600 mg/kg plus 120 mg/kg. Consequently, the above results are attributed to a possible synergism of both pesticides.

Furthermore, neonicotinoids are pesticides that act as nAChRs agonists. Several models could simulate this effect, such as the pilocarpine-induced epilepsy model. Pilocarpine is an alkaloid with cholinergic action that carries out its activities on the nAChRs, which are highly expressed in the hippocampus, known to produce seizures by increasing the activation of these receptors [30]. With dosages of 320 to 380 mg/kg of pilocarpine, animals show behavioral changes such as a fixed gaze, head oscillation, abnormal mouth movements, and salivation. Some of these behaviors were observed in the experimental assays of the present study. This model of epilepsy is developed fifteen to twenty minutes after injection. Electrographic changes are characterized by high-voltage spikes [31]. Pilocarpine-induced generalized seizures result in brain damage [32], similar to that observed after the mixture of clothianidin and thiacloprid administration at a dose of 400 and 80 mg/kg, respectively.

The histological findings made in this study demonstrate that the administration of the subchronic dose of 400/80 mg/kg of the clothianidin-thiacloprid mixture induces histological changes in the granule cells of the dentate gyrus (DG), where nucleosomal retraction and loss of tissue architecture were observed in the rats that were sacrificed 72 hours after the tenth pesticide administration. This was confirmed after a cell count, which showed a greater number of pyknotic cells in the DG of animals administered the neonicotinoid mixture, compared with the control group. These findings are similar to those reported by the administration effects of imidacloprid, which, after being administered orally in a dose of 80 mg/kg every 48 hours for 25 days, generates a decrease in the number of pyramidal and glial cells, and pycnosis in CA1 [15]. This makes it evident that neonicotinoids may be involved in neuronal death processes in this brain nucleus. The histopathological findings observed in the granule cells of the DG are possibly attributed to cellular damage by excitotoxicity, mediated by nAChRs *α*4*β*2 overstimulation, present in the glutamatergic afferents of perforating pathway [33, 34]. This performing pathway communicates with the granule cells of the DG [35], and because neonicotinoids, such as clothianidin and thiacloprid, act as nAChRs *α*4*β*2 agonists, mimicking the action of acetylcholine, it could favor the opening of ion channels and the entry of Na^+^ and Ca^2+^ into the cells [36, 37], causing excitatory overstimulation in the CNS [36]. Nowadays, there are reports that neonicotinoids, such as imidacloprid and acetamiprid, can evoke excitatory Ca^2+^ influxes in neonatal rat cerebellum granule cells [38]. In addition, exposure to imidacloprid for 28 days causes a significant elevation of calcium levels in both the hypothalamus and the pituitary gland [39]. Our study suggests that the glutamate is released from glutamatergic neurons from the entorhinal cortex, stimulated by the neonicotinoids interaction in the nAChRs *α*4*β*2, favoring the elevation of Ca^2+^ levels in the DG granular cells, due to the binding of glutamate with NMDA-type receptors (N-methyl-D-aspartate) present in these cells [40]. Likewise, we found that the histopathological findings were not easy to demonstrate and did not reveal definitive marks in the DG in the brain of rats sacrificed 19 days after the tenth administration of the neonicotinoid mixture. This is attributed to the fact that neurogenesis is a process that occurs continuously in the adult mammal DG [35, 40–43]. We deem that after having suspended the administration of neonicotinoids, the cells that died during the period of subchronic administration were replaced by newly formed granular neurons during the 19 days preceding the animal sacrifice since most progeny neural precursor cells in the DG give rise to dentate granular neurons, which differentiate and integrate into the subgranular DG layer. Between one and four weeks after their generation, developing axonal projections and generating neuritic processes happen, which allow them to integrate synaptically between two and four weeks after birth [35]. This is consistent with the hippocampus histological state in the present study, since 72 h after subchronic administration scheme and at the beginning of the tests in the MWM, the animals show a deficiency in the test of short-term memory (STM). The number of crosses made for the rats and the time spent in each quadrant were lower than those in the control group, which may be explained by the lesions in the Hp, provoking animals to have learning and memory test deficiencies in MWM [44, 45]. Likewise, deficiencies in spatial STM have been recorded in experiments performed in rats and mice after ischemic lesions in the DG [46]. Furthermore, the effect of exercise on the treadmill in gerbils with an ischemic lesion in the DG evidence showed that exercise improves the STM, suppressing apoptotic cell death induced by ischemia [47]. This result makes us believe that histological damage at this level may be closely related to our findings, which revealed animals' deficiency in the STM tests. It should be mentioned that the number of crosses and the time of permanence in the quadrant were significantly lower in the rats administered neonicotinoids. These results coincide with those reported by Özdemir and Cols (2014), who also did not observe significant differences in the test of quadrant permanence on adult rats administered different doses of clothianidin [16]. The downward trend in our results for the same test is attributed to the combined administration of neonicotinoids. It has been reported that insecticide mixtures are the most related moieties with neurotoxic effects [48].

We believed that the STM commitment in our tests would be reflected in the results obtained in the long-term memory (LTM), but it was not. This may be due to the fact that the LTM consolidation process involves several brain structures such as the hippocampal formation, including the DG, the fimbria or even the fornix and the hippocampus itself, as well as the parahippocampal gyrus, formed by the entorhinal, perirhinal, and parahippocampal cortices [49], not only the DG. Several studies propose that the mechanisms involved in STM and LTM are dissociable and that it is possible to observe deficiencies in the STM without LTM commitment [50, 51]. This is considered to occur after the subchronic neonicotinoids administration scheme, and consequently this could have a negative effect on the STM processes without repercussion in the LTM. Finally, the results obtained in the present study show the toxic effects that neonicotinoids administration can exert on the CNS, such as mild cognitive impairment, that could induce structural damage at the hippocampal level.

## Figures and Tables

**Table 1:** Determination of the subchronic administration scheme from the LD50 reported by the manufacturer.

Neonicotinoids	LD_50_ (10/10)	LD5O (10/10)	LD50 5/10	LD50 4/10	LD50 3/10	LD50 2/10	Control
Clothianidin mg/kg (Poncho® 600)	2000		1000	800	600	400	s.s.
Thiacloprid mg/kg (Calypso® 480)		400	200	160	120	80	s.s.
Death percentage (%)	100	100	100	100	100	20	0
Number of administration times	1	1	1	3	5	10	10

**Table 2:** Behavioral effect of administration of different doses of the mixture of clothianidin (Poncho® 600) and thiacloprid (Calypso® 480).

Observed signs	Control	5/10 LD_50_	3/10 LD_50_	2/10 LD_50_	Control
Decreased activity	—	x	x	x	—
Ptosis	—	x	x	x	—
Slow gait	—	x	x	x	—
Startles	—	x	x	—	—
Ataxia	—	x	x	—	—
Shrieking	—	x	x	x	—
Myoclonias	—	x	x	x	—
Prostration	—	x	x	x	—
Dyspnea	—	x	x	x	—
Seizures	—	x	x	x	—

## Data Availability

The data are completely available for analysis and verification from the corresponding author at ruomon@gmail.com and Google Drive https://drive.google.com/drive/my-drive.
